# Pulmonary Neuroendocrine Neoplasms Overexpressing Epithelial-Mesenchymal Transition Mechanical Barriers Genes Lack Immune-Suppressive Response and Present an Increased Risk of Metastasis

**DOI:** 10.3389/fonc.2021.645623

**Published:** 2021-08-30

**Authors:** Tabatha Gutierrez Prieto, Camila Machado Baldavira, Juliana Machado-Rugolo, Cecília Farhat, Eloisa Helena Ribeiro Olivieri, Vanessa Karen de Sá, Eduardo Caetano Abilio da Silva, Marcelo Luiz Balancin, Alexandre Muxfeldt Ab´Saber, Teresa Yae Takagaki, Vladmir Cláudio Cordeiro de Lima, Vera Luiza Capelozzi

**Affiliations:** ^1^Department of Pathology, University of São Paulo Medical School (USP), São Paulo, Brazil; ^2^Health Technology Assessment Center (NATS), Clinical Hospital (HCFMB), Medical School of São Paulo State University (UNESP), Botucatu, Brazil; ^3^International Center of Research/CIPE, AC Camargo Cancer Center, São Paulo, Brazil; ^4^Molecular Oncology Research Center, Barretos Cancer Hospital, Barretos, São Paulo, Brazil; ^5^Division of Pneumology, Instituto do Coração (Incor), Medical School of University of São Paulo, São Paulo, Brazil; ^6^Oncology, Rede D’Or São Paulo, São Paulo, Brazil; ^7^Department of Clinical Oncology, Instituto do Câncer do Estado de São Paulo (ICESP), São Paulo, Brazil

**Keywords:** pulmonary neuroendocrine neoplasms, epithelial to mesenchymal transition transcriptional factors, desmosomes, desmocollin, collagen, metastasis

## Abstract

Typical carcinoids (TC), atypical carcinoids (AC), large cell neuroendocrine carcinomas (LCNEC), and small cell lung carcinomas (SCLC) encompass a bimodal spectrum of metastatic tumors with morphological, histological and histogenesis differences, The hierarchical structure reveals high cohesiveness between neoplastic cells by mechanical desmosomes barrier assembly in carcinoid tumors and LCNEC, while SCLC does not present an organoid arrangement in morphology, the neoplastic cells are less cohesive. However, the molecular mechanisms that lead to PNENs metastasis remain largely unknown and require further study. In this work, epithelial to mesenchymal transition (EMT) transcription factors were evaluated using a set of twenty-four patients with surgically resected PNENs, including carcinomas. Twelve EMT transcription factors (*BMP1*, *BMP7*, *CALD1*, *CDH1*, *COL3A1*, *COL5A2*, *EGFR*, *ERBB3*, *PLEK2*, *SNAI2*, *STEAP1*, and *TCF4*) proved to be highly expressed among carcinomas and downregulated in carcinoid tumors, whereas upregulation of *BMP1*, *CDH2*, *KRT14* and downregulation of *CAV2*, *DSC2*, *IL1RN* occurred in both histological subtypes. These EMT transcription factors identified were involved in proliferative signals, epithelium desmosomes assembly, and cell motility sequential steps that support PNENs invasion and metastasis in localized surgically resected primary tumor. We used a two-stage design where we first examined the candidate EMT transcription factors using a whole-genome screen, and subsequently, confirmed EMT-like changes by transmission electron microscopy and then, the EMT-related genes that were differentially expressed among PNENs subtypes were predicted through a Metascape analysis by in silico approach. A high expression of these EMT transcription factors was significantly associated with lymph node and distant metastasis. The sequential steps for invasion and metastasis were completed by an inverse association between functional barrier created by PD-L1 immunosuppressive molecule and EMT transcriptional factors. Our study implicates upregulation of EMT transcription factors to high proliferation rates, mechanical molecular barriers disassembly and increased cancer cell motility, as a critical molecular event leading to metastasis risk in PNENs thus emerging as a promising tool to select and customize therapy.

## Introduction

Currently, neuroendocrine neoplasms (NENs) are categorized into differentiated neuroendocrine tumors (NETs), also named as carcinoid tumors (TC, typical carcinoid and AC, atypical carcinoid), and poorly differentiated neuroendocrine carcinomas (NECs), including large cell neuroendocrine carcinoma (LCNEC) and small cell lung carcinoma (SCLC) (1). Patients with PNENs have tumors sufficiently localized to be considered treatable by surgical resection, and among those whose tumors are successfully resected, approximately 90-98% of patients with typical carcinoid, and 50-60% of atypical carcinoid, survive 5 years (2, 3) even developing local invasiveness, dissemination to regional lymph nodes, and distant metastasis (4, 5) which occur in 3% of typical carcinoids and 21% for atypical carcinoids (6–8). In contrast, only 20-30% of the patients with large cell neuroendocrine carcinoma survive 5 years after surgical resection and adjuvant chemotherapy (9), and only 10% of the patients with small cell lung carcinoma survive 5 years after Cisplatin + Carboplatin + Etoposide (10).

In addition to morphological and histological differences, PNENs encompass a bimodal spectrum of metastatic tumors with differences in histogenesis. For instance, normal lung contains a population of neuroendocrine cells (NE), referred as Kulchitsky cell, within the bronchial tree and neuroendocrine bodies in the periphery, which also might give rise to carcinoids, a specific group of tumors based on their secretory products, distinct staining characteristics, and ability to uptake and decarboxylate amine precursors (11). In contrast, SCLC do not arise from Kulchitzky cells, but from multipotent or undifferentiated neuroendocrine NE cells in the central bronchial tree (12–14) as previously demonstrated in experimental models genetically modified (15). Given these unusual characteristics of PNENs, not only is it still often difficult to oncologist predicts which tumors will invade, metastasize, and abbreviate the patient’s life, nevertheless, effective adjuvant treatments still depend on identifying these tumors shortly after biopsy or surgery as well.

The tissue availability for genome investigation, looking for biomarkers that signal the risk of metastasis and cancer specific death, has primarily focused on tumor epithelial compartment (16–18) and not on their effects on molecular events for invasion more lethal, and more therapeutically relevant for metastatic lesion. Furthermore, genome-based studies that have preliminarily explored in metastatic tumors were done using small sample biopsies (19–23). Thus, the molecular mechanisms that lead to PNENs metastasis remain largely unknown and require further study. The identification of EMT-related genes in tumor epithelial compartment and their effects on metastasis steps as new biomarkers and therapeutic targets for PNENs is promising.

In order to address these gaps in the literature, we evaluated epithelial to mesenchymal transition (EMT) transcription factors, most of them involved in cancer cell proliferation signals, mechanical molecular barriers, and cell block motility that support PNENs invasion and metastasis in localized surgically resected primary tumor. Overall, we performed an analysis of EMT transcription factors expression data generated using mRNA in two approaches, where we first utilized gene expression microarray technology to identify candidate genes that are associated with PNENs metastasis, confirmed EMT-like changes by transmission electron microscopy and validation in a similar independent cohort using in silico analysis. To complete the sequential steps for invasion and metastasis by PNENs, we evaluated the functional barrier created by the PD-L1 immunosuppressive molecule.

## Materials and Methods

### Patients and Samples

#### Discovery Cohort

We analyzed tumor samples collected from 65 patients diagnosed with either a carcinoid tumor or LCNEC confirmed by surgical resection, or with SCLC confirmed by surgical resection or a biopsy, between 2007 and 2016. 24 fresh frozen tumor-normal pairs (10 SCLC, 4 LCNEC, 5 AC, and 5 TC) from A. C. Camargo Cancer Center, in São Paulo, Brazil, and Hospital do Amor, in Barretos, Brazil, and 41 archival formalin-fixed paraffin-embedded histological sections samples (13 SCLC, 9 LCNEC, 5 AC, and 14 TC) from the Hospital das Clínicas and from the Heart Institute of the University of São Paulo (USP). The neoplastic area was delimited during the frozen section procedure to ensure the exclusion of non-neoplastic tissue. At the time of resection, random samples of tumor were diced, fixed in 2.5% buffered glutaraldehyde, embedded in Araldite, and cut into thin sections that were then stained with uranyl acetate and lead citrate and examined by transmission electron microscopy (TEM) to confirm EMT-like changes. The histologic diagnosis and immunohistochemistry results were then reviewed and confirmed by two experienced lung pathologists in accordance with the WHO 2015 classification (24). The main histologic criteria for tumor re-classification were the mitotic count and the presence of an organoid pattern (rosettes, pseudo rosettes, palisading, spindle cells) or necrosis. The cohort included 29 carcinoid tumors (19 TC and 10 AC), 13 LCNEC, and 23 SCLC. Patient’s demographics and clinicopathological characteristics were obtained from medical records and included age, sex, smoking history, tumor size, tumor stage (according to the International Association for the Study of Lung Cancer classification system, 8th edition), and follow-up information (24). The internal ethics committees of all the participating institutions approved this study’s protocol (process number 1.077.100) with a waiver for informed consent by their review boards.

### Evaluation of Biological Function of EMT-Related Gene Expressions

To validate our data and to investigate which EMT biological process were involved, we conducted an interactive analysis on the Metascape^1^, a tool for gene annotation and gene list enrichment analysis, to analyze the genes whose expression differed among PNEN subtypes (25). For the gene list used, we carried out a pathway and process enrichment analysis using the following ontology sources: KEGG Pathway, GO Biological Processes, Reactome Gene Sets, Canonical Pathways, CORUM, DisGeNET. With the purpose of identifying EMT-related protein interactions, we used the Search Tool for the Retrieval of Interacting Genes/Proteins (STRING) database (26) to explore the protein-protein interaction (PPI) of the EMT-related genes that were investigated in our study. A heatmap was created to verify the association between the relative expression of the EMT transcription factors and histological subtypes using the Heatmapper platform^2^. Then, we used the average distance and the Euclidean distance between elements to perform an unsupervised hierarchical grouping.

### Epithelial-Mesenchymal Transition (EMT) Transcription Factors Identification

Total RNA was extracted from fresh-frozen tumor and normal tissues using the QIAsymphony miRNA CT 400 kit (Qiagen, CA, USA) according to the manufacturer’s instructions. RNA integrity and quality were determined using the Bioanalyzer 2100 (Agilent Technologies). Complementary DNA was synthesized using the c-DNA – RT² First Strand Kit (Qiagen Sample & Assay Technologies) according to the manufacturer’s protocol. The difference of expression in EMT genes was evaluated by the real-time PCR method. Quantitative reverse transcription-polymerase chain reaction (qRT-PCR) was performed using the RT² Profiler PCR Array System (PAHS-090Z; Qiagen, Dusseldorf, Germany) kit for the human EMT pathway with 84 target genes. The array includes a total of 84 EMT genes, 5 housekeeping genes (*ACTB*, *B2M*, *GAPDH*, *HPRT1*, *RPLP0*), 1 genomic DNA control (GDC) to assess contamination, 3 reverse transcriptase controls (RTC) that certify the efficiency of the reverse transcription step, and 3 positive PCR controls (PPC) consisting of an artificial DNA sequence certifying the test accuracy (see **Supplementary Table S1**). Each 96-well plate includes SYBR^®^ Green-optimized primer assays for a thoroughly researched panel of 84 EMT genes, that also are included the collagen and integrin genes. Furthermore, the high-quality primer design and RT^2^ SYBR^®^ Green qPCR Mastermix formulation enable the PCR array to amplify 96 gene-specific products simultaneously under uniform cycling conditions. The samples were amplified using Applied Biosystems Step One Plus (Applied Biosystems, California, USA). The cycling conditions were as follows: 95°C for 10 minutes, 40 cycles at 95°C for 15 seconds, 60°C for 1 minute, followed by the dissociation period. The data were then analyzed in the StepOne software (v. 2.0, Applied Biosystems) using the Δ threshold cycle (Ct) method (2^−ΔΔCt^) (27). All data were normalized by the housekeeping genes, and normal lung tissue specimens were used as case control. The Ct cutoff was set to 35, and Fold-Change (FC) cutoff was set to ≥2.0.

### Programmed Cell Death Ligand 1 (PD-L1) Detection

PD-L1 expression was automatically detected using the Ventana Benchmark Ultra Platform (Roche, Ventana Medical Systems Inc., Tucson, USA) with an OptiView DAB IHD Detection Kit and OptiView Amplification Kit according to proprietary protocols and using the primary anti-PD-L1 antibody SP263 (prediluted; Roche). Samples were considered positive to antigen expression at the presence of a fully membranous brownish staining.

### PD-L1 Tumor Proportion Score (TPS) Determination

The TMP of membranous PD-L1 expression in cancer cells was determined by digital image analysis. The images were captured using a Nikon camera attached to a Nikon microscope and sent to an LG monitor by means of a computer-controlled (Pentium 1330 MHz) digitalizing system (Oculus TCX, Coreco Inc., St. Laurent, Quebec, Canada). All slides were fully analyzed at a magnification of ×400 and submitted to an automatic staining vector analysis, followed by total tissue area detection, separation of tumor from non-tumor areas in each slide, and finally, automatic cellular detection. We then used a membrane algorithm to obtain the H-score of the PD-L1 membranous staining. This algorithm consisted of multiplying each staining membrane score, i.e., 0 (no staining), 1+ (weak staining), 2+ (moderate staining), or 3+ (strong staining), by the percentage of positive cell (0-100%) at that intensity to reach a final H-score ranging between 0-300 (**Figure 5**). If the H-score was equal to or higher than the mean value of all samples, PD-L1 protein expression was classified as positive, whereas an H-score lower than the mean value was classified as negative PD-L1 protein expression (28, 29).

### Data Management and Statistical Analysis

Data were collected and managed using REDCap electronic data capture tools hosted at A. C. Camargo Cancer Center, in São Paulo, Brazil, Hospital do Amor, in Barretos, Brazil, and Hospital das Clínicas and from the Heart Institute of the University of São Paulo (USP). Considering the non-normal distribution of our data, all statistical tests used in this study to examine the difference between categories and groups were non-parametric tests as follows: the chi-square test or Fisher’s exact test was used to examine differences in categorical variables, whereas either the Kruskal-Wallis test or the Mann–Whitney U test was used to detect differences in continuous variables between groups of patients. Qualitative data were described using relative frequencies. Overall survival (OS) was defined as the interval from the date of biopsy or surgical resection to death and OS curves were estimated using the Kaplan–Meier method. The Cox proportional hazards model was then used to analyze the association between OS rate and other covariances, and only parameters that presented P ≤ 0.2 in a univariate analysis were considered for multivariate analysis. We used the Statistical Package of Social Science (SPSS) version 18 for all statistical analysis. All tests with P<0.05 were deemed statistically significant and a Bonferroni correction was used when necessary.

## Results

### Functional Enrichment Analysis of EMT-Related Genes in PNENs

**Figure 1:**
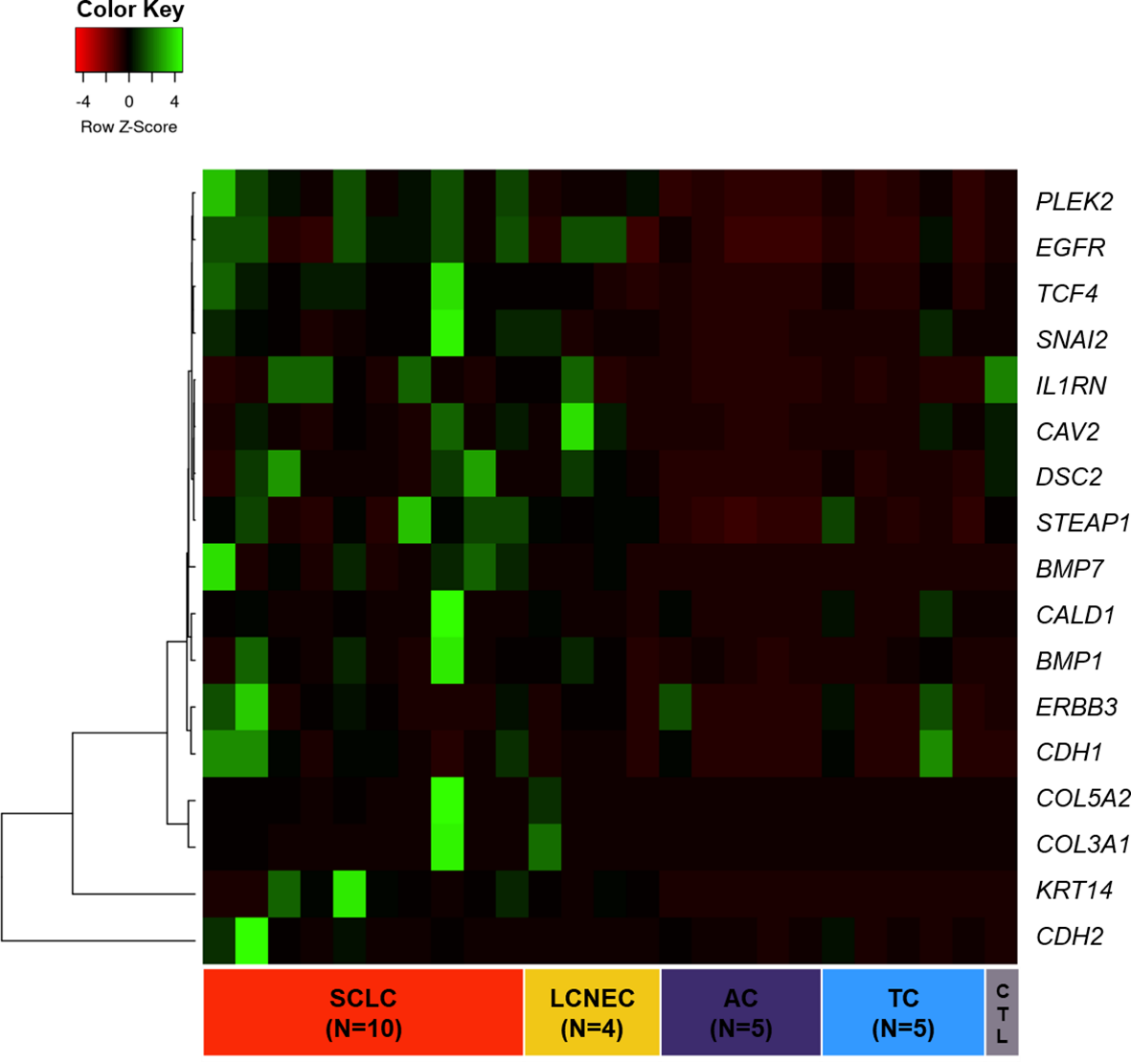
Heatmap of 17 EMT-related genes differentially expressed across PNET histological subtypes.

As shown in **Figure 1**, we first created a heatmap distribution of the 17 EMT transcription factors studied in our cohort among PNENs histological types, which showed different levels of expression (FC≥ 2.0). We observed that *BMP1*, *CDH2*, and *KRT14* were upregulated, whereas *CAV2*, *DSC2*, and *IL1RN* were downregulated in all histological subtypes. Furthermore, 12 genes (*BMP1*, *BMP7*, *CALD1*, *CDH1*, *COL3A1*, *COL5A2*, *EGFR*, *ERBB3*, *PLEK2*, *SNAI2*, *STEAP1*, and *TCF4*) were differentially expressed among histological subtypes and were all overexpressed in SCLC and LCNEC when compared to TC and AC.

**Figure 2:**
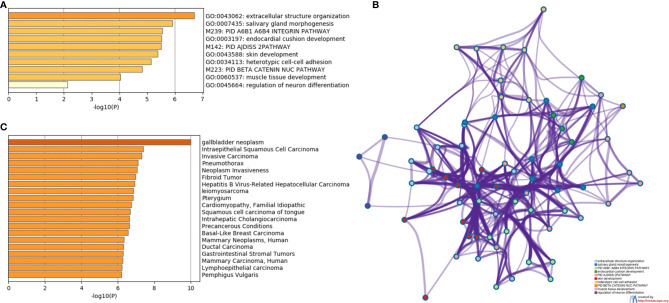
The enrichment analysis of 17 EMT-related genes using Metascape. **(A)** Heatmap of enriched terms colored by P-values. **(B)** Protein-protein interaction network. **(C)** Summary of enrichment analysis in DisGeNET colored by P-values.

**Figure 3:**
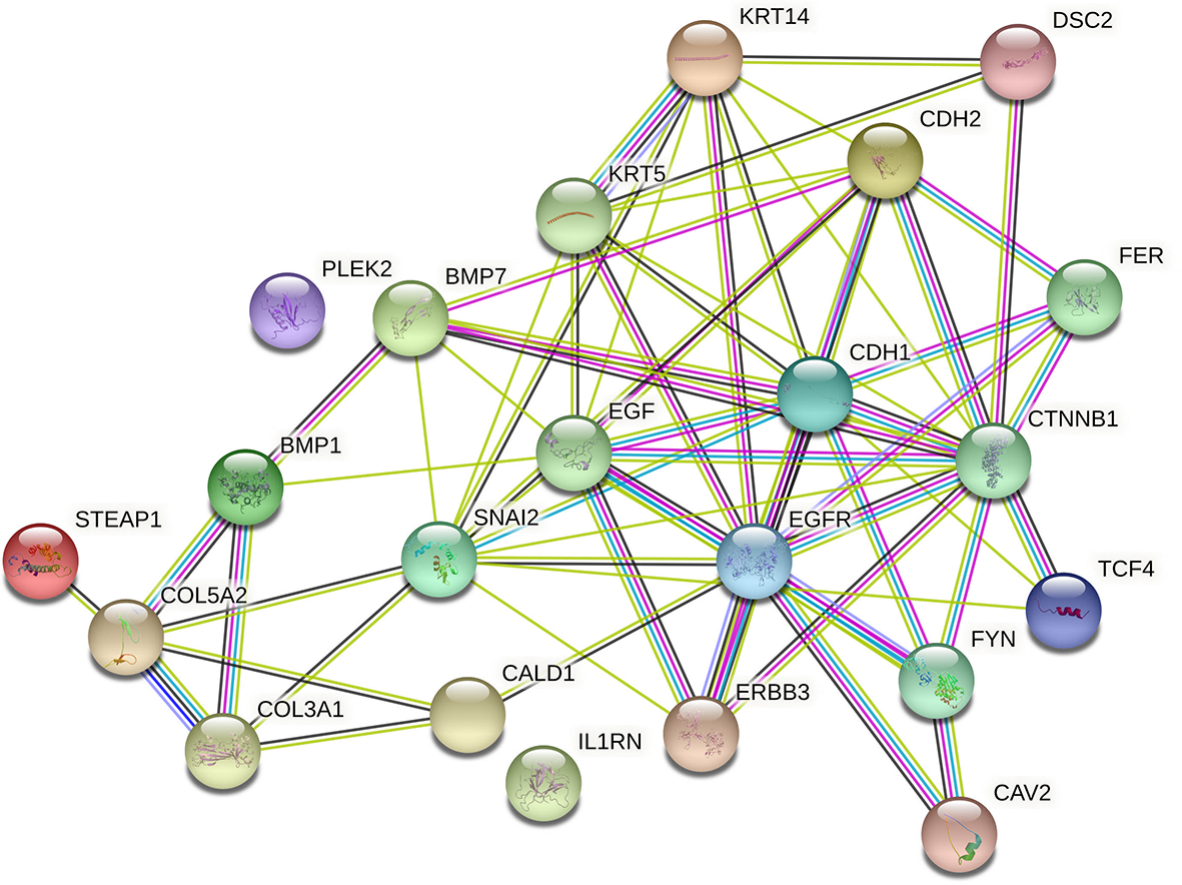
Cluster analysis of the PPI network using STRING database for EMT proteins interactions. The network included the 22 functional partners with the highest interaction confidence score, namely, STEAP1, COL5A2, COL3A1, BMP1, CALD1, IL1RN, ERBB3, SNAI2, BMP7, PLEK2, CAV2, FYN, EGFR, EGF, KRT5, KRT14, CDH1, CTNBB1, TCF4, FER, CDH2, DSC2, (score ≥ 0.9).

To assess the function and the biological process that EMT-related genes presented in our cohort, the Metascape analysis were performed. The heatmap of enriched terms across input gene lists included: “extracellular structure organization”, “salivary gland morphogenesis”, “PID A6B1 A6B4 Integrin Pathway”, “endocardial cushion development”, “PID AJDISS 2Pathway”, “skin development”, “heterotypic cell-cell adhesion”, “PID Beta Catenin NUC Pathway”, “muscle tissue development”, and “regulation of neuron differentiation” (**Figure 2A**). **Figure 2B** shows the network formed by these enriched terms. We then consulted the enrichment analysis in DisGeNET and observed that these 17 EMT-related genes are involved in many types of cancers, as shown in **Figure 2C**. Furthermore, we analyzed our gene list using PPI enrichment analyses carried out in the following databases: BioGrid6, InWeb_IM7, and OmniPath. In the resultant network, the three best-scoring terms by p-value were “degradation of the extracellular matrix”, “extracellular matrix organization”, and “extracellular structure organization”. In a second analysis, we investigated the PPI network of these EMT-related genes using the STRING database. Its molecular organization can be visualized as a network of differentially connected nodes shown in **Figure 3**. Each node stands for a protein and the edges represent dynamic interactions.

### Association Between EMT Gene Expression and Histological Subtypes

The next step was to explore the association between EMT genes and histotypes as can be appreciated in **Table 1**. We found a significant association between all histological subtypes and 11 differentially expressed EMT transcription factors, including *BMP7*, *COL3A1*, *COL5A2*, *DSC2*, *EGFR*, *IL1RN*, *KRT14*, *PLEK2*, *SNAI2*, *STEAP1*, and *TCF4* (P<0.05). It is worth noting that less differentiated tumors overexpressed of EMT genes, as is the case of SCLC when compared to TC, AC, and LCNEC, except for *DSC2* and *IL1RN* that were underexpressed in PNENs. LCNEC showed intermediate EMT gene expression, with average gene expression levels falling between that of SCLC and carcinoid tumors. TC and AC presented low expression of all the 11 EMT genes when compared to LCNEC and SCLC.

**Table 1:** Association between median EMT gene expression and histological subtypes of PNENs patients by non-parametric Kruskal-Wallis test, (P<0.05).

EMT mRNA expression	High-grade neuroendocrine tumors		Carcinoid tumors		Control	*P-value*
	SCLC	LCNEC	AC	TC	Lung normal tissue	
***BMP7***	13.39	4.23	0.41	0.78	2.14	0.003
***COL3A1***	9.46	5.05	0.59	1.18	2.97	0.006
***COL5A2***	15.54	3.24	0.64	0.65	2.58	0.005
***DSC2***	1.10	1.63	0.10	0.54	2.18	0.005
***EGFR***	9.26	6.90	0.10	0.78	3.09	0.036
***IL1RN***	0.49	0.49	0.07	0.04	2.57	0.009
***KRT14***	112.77	76.11	10.55	2.32	2.00	0.010
***PLEK2***	11.87	3.97	0.25	1.13	2.02	0.002
***SNAI2***	4.35	2.17	0.38	1.09	2.18	0.005
***STEAP1***	2.72	2.67	0.67	1.34	2.40	0.010
***TCF4***	7.74	3.86	0.33	0.65	2.58	0.004

PNENs, pulmonary neuroendocrine neoplasms; TC, typical carcinoid; AC, atypical carcinoid; LCNEC, large cell neuroendocrine carcinoma; SCLC, small cell lung carcinoma.

### EMT-Like Changes by Transmission Electron Microscopy (TEM)

**Figure 4:**
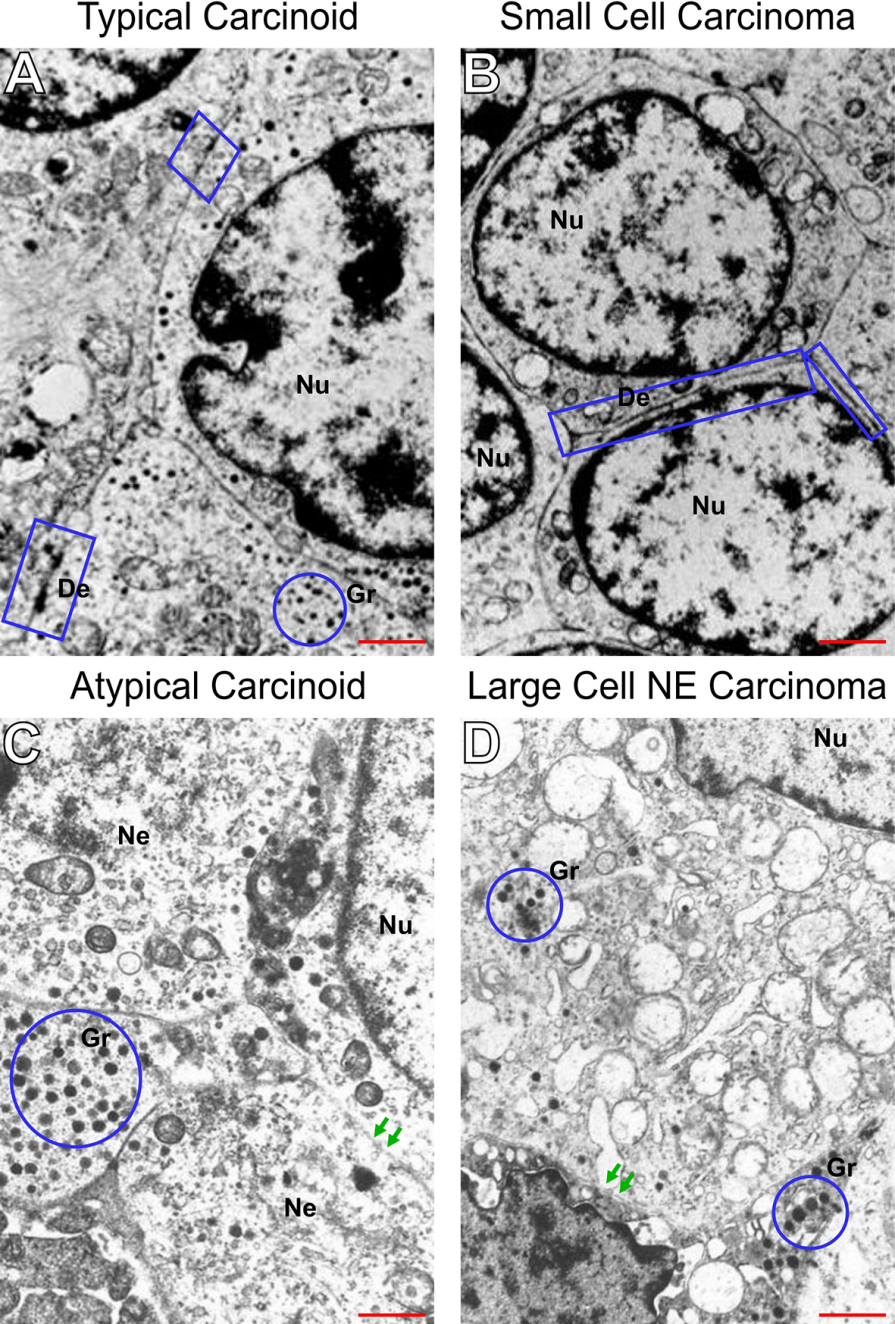
Disruption of intercellular junctional complexes of PNENs cells and enhanced breakdown of the basement membrane. Transmission electron micrographs of PNENs cells in typical carcinoid **(A)**, small cell lung carcinoma **(B)**, atypical carcinoid **(C)**, and large cell neuroendocrine carcinoma **(D)**. Ultrastructural, the TC and SCLC present nucleus with condensed chromatin on the periphery, whereas in AC and LCNEC the nucleus are more vesiculous with sparse distribution of chromatin. The neuroendocrine origin of the four subgroups is evident by different amounts of the NE granules (blue circles) inside of cytoplasmic processes or dispersed in the cytoplasm. In SCLC, the tumor cells were loosely dissociated with each other through tenues desmosomes compared to TC (the electron-dense materials at the lateral side, blue circle). Note that the intercellular junctional complex are not prominent in SCLC compared to TC, suggesting abnormal levels of desmocolin. In contrast, diminishing of electron-dense materials, indicating disruptions of intercellular junctional complexes, and enhanced breakdown of the basement membrane indicating the opening of cell-cell contacts in AC and LCNEC (red arrows). The micrographs are representative PNENs sections from 24 patients. Scale bars: 1 mm. De, desmosomes; Nu, nucleus; Gr, granules; Ne, neuroendocrine.

**Figure 5:**
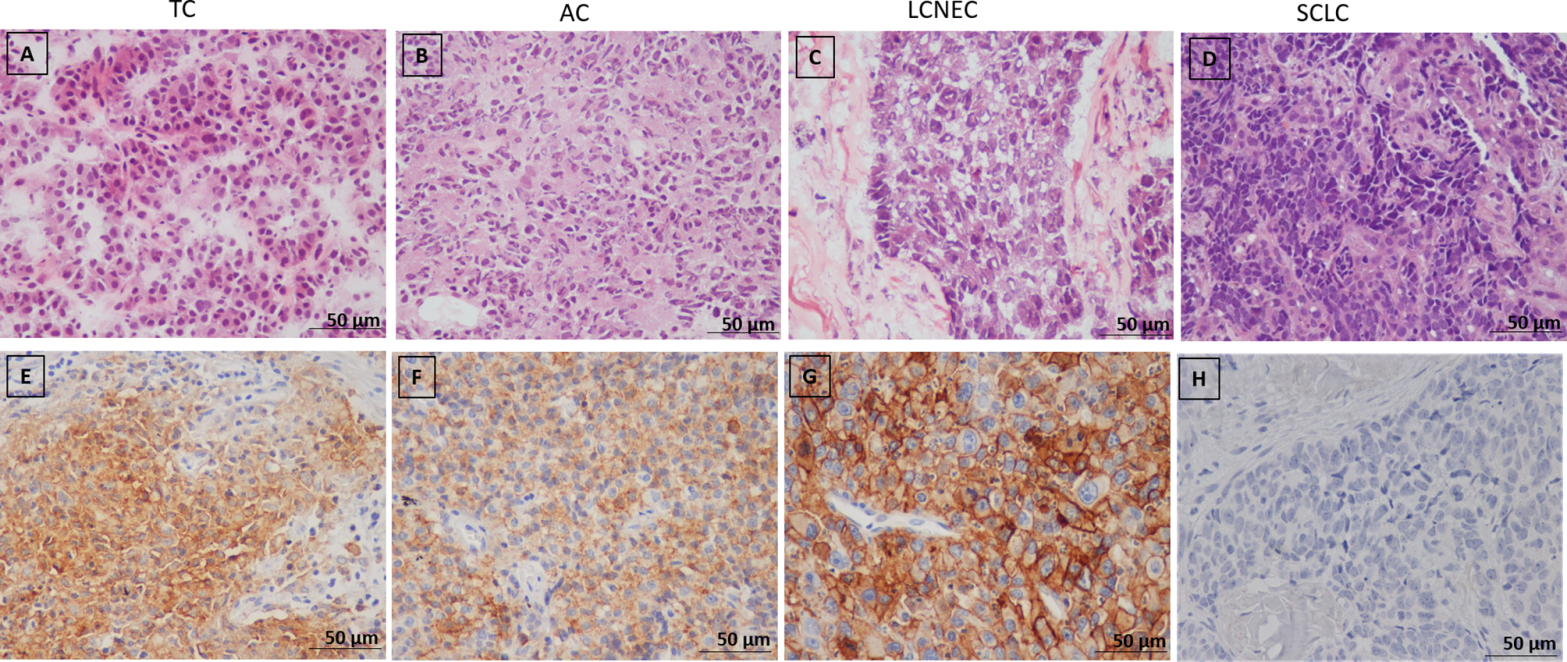
**(A–D)** Representative microphotographs of pulmonary neuroendocrine neoplasms subtypes (H&E), **(A)** Typical carcinoid (TC), **(B)** Atypical carcinoid, **(C)** Large cell neuroendocrine carcinoma (LCNEC), **(D)** Small cell lung carcinoma (SCLC); **(E–H)** Immunohistochemical staining of PD-L1 in PNENs, **(E, F)** Moderate PD-L1 immunostaining in typical (TC) and atypical carcinoid (AC), respectively; **(G)** Strong PD-L1 immunostaining in large cell neuroendocrine carcinoma (LCNEC), **(H)** Negative PD-L1 immunostaining in small cell lung carcinoma (SCLC).

Subsequently, we examined the PNENs cancer cells by transmission electron microscopy and optical microscopy to find morphologic evidence to correlate with the molecular changes, as shown in **Figure 4**. In TC and AC (**Figures 4A, C**, respectively) the cancer cells were intimately associated with each other through desmosomes located in the lateral membrane although they showed a tenuous disassembly. In contrast, SCLC and LCNEC (**Figures 4B, D**, respectively) showed an intense loss of desmosome morphology and disassembly of cell-cell contacts. The ultrastructure changes revealing mechanical barriers disassembly coincided with loss of cohesiveness between cancer cells visualized at hematoxylin-eosin in PNENs (**Figures 5A–D**).

### Association Between PD-L1 Signal and Clinicopathological Features

The next step was to investigate whether PD-L1 functional immune suppressive barrier was also compromised in these tumors (**Figure 5**). In fact, the observation of a negative signal intensity of PD-L1 was associated with high malignant potential tumors, such as SCLC (**Figure 5H**). Furthermore, membranous signal intensity of PD-L1 increased progressively as tumor malignancy, being higher in LCNEC, lower in AC, and lowest in TC (**Figures 5E–G**, respectively). These findings coincided with a significant difference in PD-L1 H-score signal intensity in PNEN tumor cells (P<0.01). A PD-L1 H-score < 1.021 was detected in 13 TC (68.4%), 4 AC (44.4%) and 4 LCNEC (33.3%), whereas a PD-L1 H-score > 1.021 was found in 6 TC (31.6%), 5 AC (55.6%), and 8 LCNEC (66.7%). Moreover, when the Kruskal-Wallis test was performed, a significant correlation emerged between PD-L1 expression and histological types (P=0.0001). In addition, PD-L1 H-score > 1.021 was significantly associated with advantageous clinicopathologic parameters, such as early stage (I and II) (P=0.03) and N0 status (P=0.02) (**Table 2**).

**Table 2:** Correlation between PD-L1 expression in neuroendocrine tumor cells and clinicopathological characteristics of PNENs patients, by Mann-Whitney test, (P<0.05).

Variables	Neuroendocrine tumor cells PD-L1 Expression (mean)	*P*-value*
**Gender**		
* Male*	0.71	0.30
* Female*	1.33	
**Age, median (years)**		
*< 58*	0.34	**0.01**
* ≥ 58*	1.80	
**Smoking Status**		
* Yes*	0.55	0.80
* No*	0.77	
**Histological subtypes**		
* CT*	1.02	0.05
* LCNEC*	2.83	
**Clinical Stage** ^†^		
* I-II*	0.80	**0.03**
* III-IV*	0.001	
**Lymph node Metastasis**		
* Yes*	0.5	**0.02**
* No*	1.97	

CT, carcinoid tumors; LCNEC, large cell neuroendocrine carcinoma; PD-L1, programmed death-ligand 1.

^†^8th Edition International Association for the Study of Lung Cancer (24).

^*^Bolded values refers to a statistical significance of p-value (P<0.05).

Of note, we observed a significant inverse association between PD-L1 H-score and the expression of *COL5A2* (R= -0.45; P=0.03), *DSC2* (R= -0.51; P=0.01), *KRT14* (R= -0.46; P=0.02), *IL1RN* (R= -0.53, P=0.01), and *STEAP1* (R= -0.45; P=0.03) by a nonparametric Spearman’s rank correlation coefficient. These associations confer an opposite effect between EMT and PD-L1 and might influence the neoplastic cells to control invasion and tumor metastasis.

### Association Between EMT Gene Expression and Clinical Characteristics of PNENs Patients

Last but not least, the functional and mechanical molecular barriers players intensifies the clinical scenario of the theatrical course of PNENs. The clinical characteristics of the patients enrolled in our study are summarized in **Table 3**. Patients had a median age at diagnosis of 58 years and were evenly distributed between genders, 34 female and 30 males. Most of the patients were diagnosed at the early stage of disease (32/29) and were smokers (38), mainly in SCLC (21). However, in our cohort, of the 19 TC patients, 9 were smokers, contradicting the fact that in majority of the cases, these patients are never-smokers. Since smoking history was obtained from the patients’ electronic medical records, this discrepancy was observed and that somehow needs to be proven in the current studies that we are conducting. Fourteen patients of our cohort developed distant metastasis. The median follow-up of the patients was 36 (0–100) months.

**Table 3:** Frequency of demographic and clinical characteristics of PNENs patients.

Characteristics	Number of Patients (N=65)
^a^ **Age, years**	
*Median (range)*	58 (19-80)
<58	34 (52.3%)
≥58	30 (46.2%)
**Sex**	
*^a^Male*	30 (46.2%)
* Female*	34 (52.3%)
^a^ **Smoking status**	
* Yes*	38 (58.5%)
* No*	20 (30.8%)
**Histological subtype**	
* SCLC*	23 (35.4%)
* LCNEC*	13 (20.0%)
* AC*	10 (15.4%)
* TC*	19 (29.2%)
^a^**TNM stage**†	
* I/II*	32 (49.2%)
* III/IV*	29 (44.6%)
^a^ **Distant metastasis**	
M0	32 (49.2%)
M1	14 (21.5%)
**Follow-up** (months)	36 (0-100)

PNENs, pulmonary neuroendocrine neoplasms; TC, typical carcinoid; AC, atypical carcinoid; LCNEC, large cell neuroendocrine carcinoma; SCLC, small cell lung carcinoma.

a Some cases lacked follow-up information: gender (1); age (1); smoking status (7); TNM stage (4); Distant metastasis (19).

We observed statistical differences between the expression of some EMT genes (*BMP1*, *BMP7*, *COL3A1*, *CDH1*, *EGFR*, *EBB3*, *PLEK2*, and *TCF4*) and the clinical stage of patients, especially at the presence of overexpression of these genes. Patients with advanced stage (III/IV) at diagnosis presented tumors that significant overexpressed *BMP1* (61.9%, P=0.042), *BMP7* (47.6%, P=0.008), *CDH1* (57.1%, P=0.002), *COL3A1* (52.4%, P=0.002), *EGFR* (47.6%, P=0.008), *ERBB3* (61.9%, P=0.001), *PLEK2* (57.1%, P=0.003), and *TCF4* (57.1%, P=0.003) (see **Supplementary Figure S1**). Patients with a smoking history also presented a significant difference in gene expression. Their tumors overexpressed *BMP7* (43.5%, P=0.027), *COL3A1* (47.8%, P=0.009), *EGFR* (43.5%, P=0.027), *PLEK2* (56.5%, P=0.001), and *SNAI2* (47.8%, P=0.039) (see **Supplementary Figure S2**). We also found a significant association between patients with lymph node metastasis and overexpression of *BMP7* (47.4%, P=0.005), *CALD1* (42.1%, P=0.020), *CDH1* (52.6%, P=0.001), *COL3A1* (42.1%, P=0.020), *EGFR* (47.4%, P=0.005), *ERBB3* (57.9%, P=0.000), *PLEK2* (47.4%, P=0.024), and *TCF4* (52.6%, P=0.001) (see **Supplementary Figure S3**). Finally, patients with distant metastasis had tumors that overexpressed *COL3A1* (38.9%, P=0.013), and *COL5A2* (38.9%, P=0.038) (see **Supplementary Figure S4**). We found no statistical differences between these genes and adjuvant treatment, age, or gender in our cohort.

### Survival Analysis

**Figure 6:**
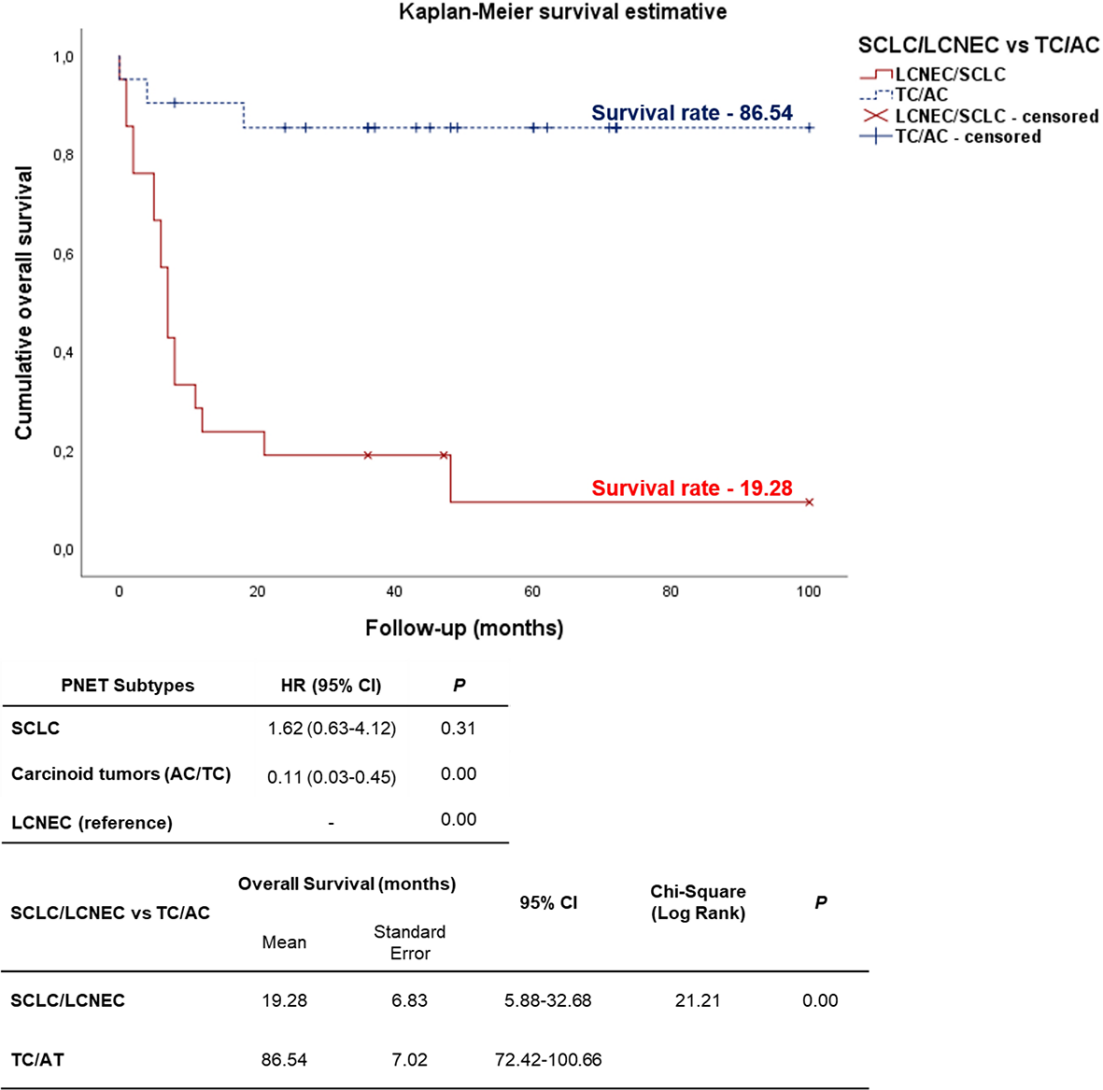
Kaplan-Meier curve according to the PNENs histological subtypes. Patients diagnosed with carcinoid tumors (TC/AC) appears as top curve (median survival 86.54 months), while those who were diagnosed with high-grade neuroendocrine tumors (SCLC and LCNEC) (bottom curve) had median survival time of 19.28 months (P<0.01 by log-rank test).

As expected, the cumulative survival rate of patients stratified by PNEN morphologic types was significantly higher in carcinoid tumors (86.54 months) than in the LCNEC and SCLC (19.28 months) (log-rank = 21.21, P<0.01) (**Figure 6**). In the Cox univariate analysis, the following variables were significantly associated with low risk of death: tumor T1 stage, N0 stage, M0 stage, and early clinical stage. The down-expression of *CDH1*, *COL3A1*, *DSC2*, *EGFR*, *PLEK2*, and *TCF4* was also associated with a low risk of death (**Table 4**, P<0.01).

**Table 4:** Variables associated with overall survival (OS) in PNENs patients.

	Univariate Analysis			Multivariate Analysis	
	HR (95% CI)	HR	*P*-value	HR (95% CI)	*P-*value*
**Clinicopathological Characteristics**					
Age, median (yrs): <58 *vs* ≥58	0.60 (0.25-1.41)	0.52	0.24		
Gender					
* Male*	1.69 (0.71-4.00)	0.52	0.23		
Smoking status					
* Yes*	4.78 (1.38-16.50)	1.56	**0.01**		
T Stage (Tumor invasion)^†^					
* T1*	0.16 (0.04-0.66)	-1.77	**0.01**		
* T2*	0.51 (0.16-1.63)	0.65	0.26		
* T3*	0.46 (0.05-3.80)	0.76	0.47		
* T4 (reference)*			0.08		
Lymph Node Status (N)^†^					
* N_0_*	0.12 (0.03-0.47)	-2.10	**0.00**		
* N_1_*	0.43 (0.10-1.83)	0.83	0.25		
* N2*	0.99 (0.26-3.78)	0.00	0.99		
* N3 (reference)*			**0.00**		
M stage (Distant Metastasis)^†^					
* Absence*	0.32 (0.12-0.84)	-1.12	**0.02**		
Clinical stage					
* Early (I/II)*					
* Advanced (III/IV) (reference)*	0.16 (0.06-0.43)	-1.81	**0.00**		
Histological subtypes					
*SCLC*	1.62 (0.63-4.12)	0.48	0.31	3.75 (0.54-25.91)	0.17
	0.11 (0.03-0.45)	-2.12	**0.00**	0.03 (0.001-0.91)	**0.04**
*Carcinoid tumors (AC/TC)*					
*LCNEC (reference)*			**0.00**		**0.01**
**PD-L1 protein expression (mean)**					
< 1.021	1.35 (0.31-5.84)	0.30	0.68		
≥ 1.021 *(reference)*					
**EMT gene expression (median)**					
***CDH1* mRNA**					
< 2.82 (N=8)	0.06 (0.008-0.53)	-2.71	**0.01**		
≥ 2.82 (N=15) *(reference)*					
***COL3A1* mRNA**					
< 2.97 (N=11)	0.17 (0.04-0.65)	-1.72	**0.00**	0.99 (0.97-1.02)	0.81
≥ 2.97 (N=13) *(reference)*					
***DSC2* mRNA**					
< 2.18 (N=18)	0.16 (0.04-0.59)	-1.79	**0.00**	1.05 (0.82-1.34)	0.66
≥ 2.18 (N=3) *(reference)*					
***EGFR* mRNA**					
< 3.09 (N=12)	0.25 (0.07-0.83)	-1.36	**0.02**	1.07 (0.88-1.31)	0.46
≥ 3.09 (N=11) *(reference)*					
***PLEK2* mRNA**				0.82 (0.69-0.98)	**0.02**
< 2.02 (N=9)	0.14 (0.03-0.68)	-1.92	**0.01**		
≥ 2.02 (N=14) *(reference)*					
***TCF4* mRNA**				1.09 (0.86-1.38)	0.44
< 2.58 (N=9)	0.13 (0.03-0.65)	-1.97	**0.01**		
≥ 2.58 (N=14) *(reference)*					

Univariate and multivariate analysis employed a Cox proportional hazards model. Chi-square 24.16, P=0.001.

HR, hazard ratio (β coefficient); CI, confidence interval; PD-L1, Programmed death-ligand; EMT, epithelial-to-mesenchymal transition.

^†^8th Edition International Association for the Study of Lung Cancer (24).

^*^Bolded values refers to a statistical significance of p-value (P<0.05).

One case lacked follow-up information about survival.

**Figure 7:**
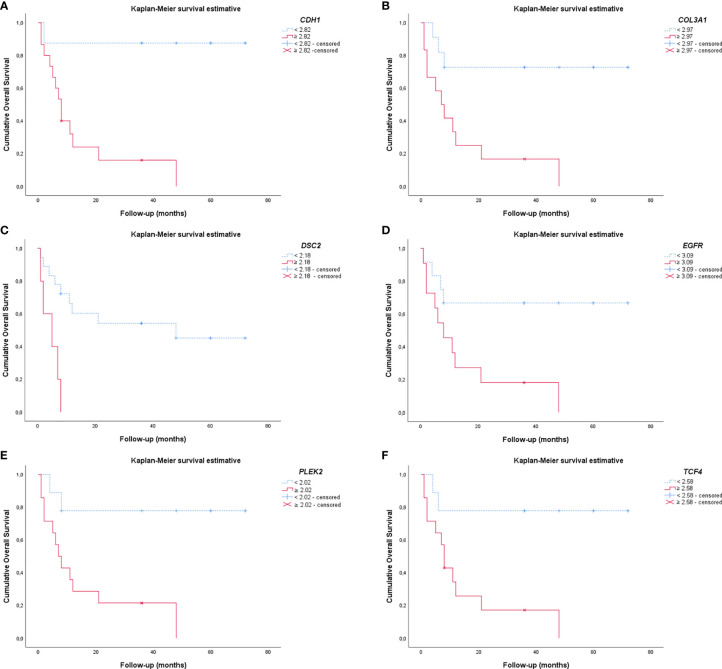
Kaplan-Meier curves show the difference in EMT gene expression between patients regarding the PNENs variants and the risk of death. **(A)** *CDH1*; **(B)** *COL3A1*; **(C)** *DSC2*; **(D)** *EGFR*; **(E)** *PLEK2* and **(F)** *TCF4*. For all these EMT genes, patients that had lower expression appeared as the top curve, and their median survival time ranged between 40-63 months. In contrast, patients with higher expression of these genes (bottom curve) had a median survival time range of 4-15 months (P ≤ 0.01 by log-rank test).

EMT gene expression was different between patients of different PNEN variants, who showed distinctly different average survival times in our Kaplan-Meier curve. The group with lower expression of *CDH1*, *COL3A1*, *DSC2*, *EGFR*, *PLEK2*, and *TCF4* (top curve) had a median survival time between 40-63 months. By contrast, those with higher expression of these genes (bottom curve) had a median survival time between just 4-15 months (P ≤ 0.01), as determined by log-rank test (**Figure 7**). Once these variables were accounted for in a multivariate analysis, where the mathematical model was controlled by histology, we found that patients who presented low expression of *COL3A1*, *DSC2*, *EGFR* and *TCF4* as co-dependent variables, as well as low expression of *PLEK2* as an independent variable, were at low risk of death. The chi-square including the covariates was 24.16, (P<0.01).

## Discussion

We evaluated EMT-related genes, using a set of twenty-four patients with surgically resected PNENs, including SCLC. These EMT transcription factors were involved in proliferative signals, epithelium desmosomes assembly, and cell motility that support PNENs sequential steps for invasion and metastasis in localized surgically resected primary tumor. We used a two-stage design where we first examined the candidate EMT genes using a whole-genome screen, and subsequently, we analyze the upregulation of these genes through Metascape analysis *in silico* approach. Additionally, high expression of EMT genes involved in cellular proliferation, epithelium desmosomes barrier, and cell motility were significantly associated with lymph node metastasis, and distant metastasis. To complete the sequential steps for invasion and metastasis by PNENs, we evaluated the PD-L1 immune checkpoint status and found an inverse association between EMT genes and PD-L1 expression. Our findings suggest that incorporation of EMT-related gene expression profile to routine genome-wide examination of biomarkers helps to predict metastasis in PNENs and may be a promising tool to select and customize therapy.

Over the past decade, treatment options for metastatic PNENs have increased, although mortality and 5-year survival remain little altered for PNETs (30) and PNECs (31, 32). Molecular studies identified somatic mutations, somatic copy numbers, and pathway alterations in primary PNENs tumors (16–18); however, less is known regarding the steps of the molecular events for invasion and metastasis by PNENs, more lethal and therapeutically relevant. It is worth emphasizing that PNENs, unlike other lung tumors, are solid tumors basically composed of highly cohesive epithelial cells intermingled by thin connective tissue septa and thin-walled vessels with a limited number of immune cells. In this context, the matricellular and vascular structure of the adjacent lung parenchyma represents the scenario for invasion. Therefore, studies that interrogate genes in tumors associated with cohesiveness of neoplastic cells, and their motility, are critical to understanding the biology of invasion and metastasis in these tumors, the major cause of patient mortality.

The process of cancer cell invasion and metastasis undoubtedly comprises a series of complex multistep process in which phenotype differences are mediated by a network of transcription factors. Among these, the EMT process signalized by tumor cells is thought to be important because facilitates cancer cell detachment, motility and penetration into blood and lymphatic vessels. Thus, a clear knowledge of EMT underlying molecular mechanism is crucial for effective targeted therapies. Despite extensive efforts, the regulatory mechanism of EMT in most cell/tissue types is not fully resolved as in case of the complex PNENs (33, 34). In order to understand the roles of EMT transcription factors in metastatic process, we explored the mRNA level in PNENs. Overall, we observed that the EMT-related genes were upregulated in SCLC, downregulated in carcinoid tumors, and presented an intermediate level of expression in LCNEC. In fact, the expression of EMT genes was much lower in carcinoid tumors when compared to SCLC and LCNEC, offering a plausible explanation for their progressive changes in phenotype and clinical spectrum, discussed below.

We found that EMT-related genes including cadherin, desmocollin, collagen, and tyrosine kinase receptors were overexpressed in PNENs. Actually, Gene Ontology and functional enrichment analysis confirmed that these highly enriched and overrepresented genes are implicated in cellular and extracellular barriers to mediate motility, invasion, and metastasis. In agreement with other studies, our results support the notion that the synergistic action of these EMT master transcription factors, functioning as invasion oncogenes in PNENs (35–37).

Notably, by transmission electron microscopy we showed that desmosomes disassembly barrier intensity was the expression phenotype factor that coincided with the levels of EMT genes encoding six barrier molecules, including *BMP1*, *BMP7*, *CALD1*, *KRT14*, *CAV2*, *IL1RN*, and three adherent junctions proteins (CDH1, CDH2, DSC2), associated to risk of lymph node and distant metastasis in PNENs. Accordingly, we have found that PNETs and PNECs expressed high levels of *BMP1*, *CDH2*, and low levels of *CAV2*, *DSC2*, *IL1RN*, whereas *BMP7*, *CALD1*, and *CDH1*, were overexpressed by PNECs and low expressed by PNETs, suggesting a positive autogenous transcriptional regulation involving activation of the EMT-related genes by phosphorylation (38) to maintain the mechanical barriers between neoplastic cells. Actually, barriers can be mediated through both tight junctions and desmosomal adhesion. Desmosomes, or macula adherens, are intermediate filament-based cell–cell adhesions using desmosomal cadherins anchored to intermediate filaments *via* desmoplakins. Expression of desmosomal barrier molecules has been observed in several solid tumors, with mixed prognostic associations. In melanoma, elevated levels of the cadherin desmocollin 3 (DSC3) has been associated with increased metastatic risk, but in colon and lung cancer, it has been associated with a better prognosis (39–41).

Remarkably, we have also found that the expression of PD-L1, which has an immunosupressive function in the anti-tumor immune reaction, correlated inversely with EMT transcription factors related to barriers (*CDH2*, *CAV2*, *DSC2*, and *IL1RN*). This inverse relationship was previously demonstrated in melanoma and ovary cancer in a well-designed study done by Salerno and colleagues (42). According to those authors, physical or mechanical barriers created by endothelial or epithelial cells with tight junctions, or functional barriers created by immunosuppressive molecules including PD-L1 (43) can also limit the immune cell infiltration valued as immune escape by tumors which can otherwise be targeted effectively with immunotherapy (44). Here, we provide two important evidence. First, carcinoids and large cell neuroendocrine carcinomas can express functional barriers created by immunosuppressive PD-L1 thus limiting the immune cell infiltration to reward the low expression of the desmosomes disassembly barrier, justifying the longer survival of the patients even with metastasis. Second, small cell lung carcinomas are associated with the lack of PD-L1 immunosuppressive barrier allowing the immune cell infiltration, but with high expression of desmosomes disassembly barrier, both facilitators of distant metastasis and shorter overall patient survival, otherwise be targeted effectively with immunotherapy PD-L1 expression. Third, the high levels of EMT transcriptions factors expressed by SCLC cells can confer mesenchymal properties, on the one hand justifying its histologic fusiform pattern, and on the other hand the acquisition of immune regulatory capacities for checkpoint blockade immunotherapy, as recently demonstrated in an elegant work made by Kursunel and colleagues (14).

Our current understanding of EMT may also be associated to the established transcriptional regulators that are classically studied separately. Therefore, the synergistic action among cooperative transcriptional factors, which has emerged as a general characteristic of enhancers (45), has not been included in the transcriptional programs of EMT. In sharp divergence to the established EMT model, in which a single transcriptions factor such as *SNAI2* controls the EMT program, we identified other master transcriptional factors suggesting a synergistical control of the EMT transcriptional program in metastatic behavior of PNENs, reinforcing the idea that EMT is a phenomenon highly dependent on the PNENs cellular machinery in which it occurs.

String database analysis confirmed the PPI network of these EMT-related genes, its molecular organization as a network of differentially connected nodes, and dynamic synergism. We found that *EGFR* and *ERBB3* were also upregulated in PNECs and downregulated in PNETs and may synergistically determine a positive feedback with desmosomes disassembly barrier. This finding gains strength in the literature by demonstrating that inhibition of *EGFR* promotes desmosome assembly by upregulation of desmosomal proteins such as desmoglein 2 and desmocollin 2 in squamous cell carcinoma of head and neck (46). In another study, activation of *EGFR* led to decreased protein levels of *DSC3*, whereas *EGFR* inhibition resulted in enhanced expression of *DSC3*, indicating an *EGFR*-dependent regulation of *DSC3* in lung cancer. Interestingly, these authors also found the inhibition of *EGFR* by gefitinib increased the expression of *DSC3* (39).

Importantly, EMT genes are involved in crucial processes, such as cellular development and differentiation, cellular growth and proliferation, cell migration and motility, and extracellular matrix invasion and cellular adhesion. Regarding *TCF4* transcription factor, it has been reported that it plays a key role in the initiation of colorectal cancer progression through the upregulation of β-catenin/*TCF4* target genes, such as c-*MYC*, *AXIN2*, and *LGR5*, regulating cell proliferation and stemness in epithelial stem and progenitor cells (47). In lung adenocarcinoma, *STEAP1* is reported to be involved in processes closely associated with cancer cell proliferation, such as cell division, cytokine production, cytokine signaling, and DNA replication (48). In addition, *PLEK2* interacts with the actin cytoskeleton to induce cell spreading, whereas *IL1RN* modulates immune and inflammatory responses, and *SNAI2* facilitate microenvironment invasion and dissemination (49, 50). However, there is limited knowledge of the role of these EMT-related genes in neuroendocrine tumors.

In our cohort, EMT genes as *COL3A1*, *COL5A2*, *PLEK2*, and *SNAI2* presented high expression in PNECs and low expression in PNETs. As PNENs are tumors with a limited stroma, the question is how the tumor cells migrate to gain access into vessels? Recently, mechanical properties of tissue have growing sympathy for tissue stiffness, defined as the resistance to deformation in response to applied force, in a multitude of biological processes (51). Gene expression is controlled by spatial and temporal variability of tissue stiffness and, ultimately, determine the differentiation lineages of stem cells (52). Not least important, different gradients in tissue stiffness works as powerful signal to guide migrating cells during cancer dissemination (53, 54).

Therefore, we speculate that neuroendocrine cancer cells transformed by EMT process acquire stem-like and mesenchymal properties to upregulate tissue stiffness by increasing ECM deposition facilitating motility and permeation in vessels. This powerful feedforward loop raises numerous possibilities for drug development and warrants further investigation into the mechanisms specific to different PNENs.

Taken together, the molecular and ultrastructural events described here may justify the progressive changes in phenotype and clinical spectra of PNENs. The dysregulated expression of *BMP1*, *BMP7*, *CALD1*, *KRT14*, *CAV2*, *IL1RN*, and adherent junctions proteins (CDH1, CDH2, DSC2), promotes the primordial detachment between the distal cancer cells, depriving the central cancer cells of the blood supply leading to comedonecrosis in AC and LCNEC and “geographic” in SCLC. *EGFR* and *ERBB3* were also upregulated in PNECs and downregulated in PNETs promoting the cancer cell proliferation, justifying the progressive increase of Ki67 antigen detected within the nucleus during interphase, and increased mitosis index after the protein relocation to the surface of the chromosomes. In this context, the Ki-67 protein increases during all active phases of the cell cycle (G1, S, G2, and mitosis) from TC to SCLC.

Evidently, these molecular biomarkers may justify the differences between the clinicopathologic features and survival analysis of SCLC/LCNEC and carcinoid tumors. In fact, a Cox multivariate analysis showed that when the mathematical model was controlled by histology, patients that presented low expression of *COL3A1*, *DSC2*, *EGFR*, and *TCF4*, as co-dependent factors, and low expression of *PLEK2*, as an independent factor, lead to a significantly low risk of death and better survival in PNENs patients.

In summary, the results presented herein provide important molecular evidence that EMT-related genes are involved in cancer cell proliferative signals, desmosomes disassembly, and cell motility that support PNENs sequential steps for invasion and metastasis in localized surgically resected primary tumor. Specifically, our study indicates that PNENs overexpressing EMT-mechanical molecular barriers, genes lack functional immune suppressive barrier and present increased patient mortality risk due to metastasis and thus potentially offer insight into novel therapeutic targets. Overall, these EMT genes may represent partially an EMT ‘remodeling’ program to drive metastatic establishment.

## Data Availability Statement

The original contributions presented in the study are publicly available. This data can be found here: https://www.ncbi.nlm.nih.gov/geo/query/acc.cgi?acc=GSE181381.

## Ethics Statement

The study was approved in accordance with the ethical standards of the responsible committee on human experimentation local (Research Ethics Committee of University of São Paulo Medical School - CAAE:17436113.9.0000.0068; opinion number: 1.0077.100) and with the 1964 Helsinki declaration. A waiver of the requirement for informed consent was obtained from committee, and to identity of the subjects under this retrospective analysis was omitted and anonymized. Written informed consent for participation was not required for this study in accordance with the national legislation and the institutional requirements.

## Author Contributions

Conception and design: VLC and TP. Writing, review, and editing: VLC, TP, CB, and JM-R. Data analysis and interpretation: VLC, TP, and CB. Statistical analysis: CF, VLC, and TP. Provision of study materials or patients: MB, AA, ES, EO, VS, and TT. Administrative support: VLC. All authors contributed to the article and approved the submitted version.

## Funding

This work was supported by Sao Paulo Research Foundation (FAPESP; 2018/20403-6, 2019/12151-0) and the National Council for Scientific and Technological Development (CNPq; 483005/2012-6). The SP263 antibody was granted as courtesy by Roche Diagnostics (Roche-Ventana, Tucson, USA).

## Conflict of Interest

The author VCL was employed by the company Rede D’Or São Luiz S.A.

The remaining authors declare that the research was conducted in the absence of any commercial or financial relationships that could be construed as a potential conflict of interest.

## Publisher’s Note

All claims expressed in this article are solely those of the authors and do not necessarily represent those of their affiliated organizations, or those of the publisher, the editors and the reviewers. Any product that may be evaluated in this article, or claim that may be made by its manufacturer, is not guaranteed or endorsed by the publisher.

## Footnotes

1
https://metascape.org/

2
http://www.Heatmapper.ca/expression/

## Supplementary Material

Supplementary Figure 1 Box plot of the associations between EMT gene expression and Clinical stage – Early (I/II) *vs* Advanced (III/IV) in a log scale. The top and bottom of the box plot represents the 25th and 75th percentile range. The line across the box shows the median of gene expression and the top and bottom bars show the maximum and minimum values, outliers were showed. The association between EMT gene expression and Clinical stage was calculated by Fisher’s exact test. **(A)** *BMP1*mRNA (P=0.042); **(B)** *BMP7*mRNA (P=0.008); **(C)** *CDH1*mRNA (P=0.002); **(D)** *COL3A1*mRNA (P=0.002); **(E)** *EGFR* mRNA (P=0.008); **(F)** *ERBB3*mRNA (P=0.001); **(G)** *PLEK2*mRNA (P=0.003) and **(H)** *TCF4*mRNA (P=0.003).

Supplementary Figure 2 Box plot of the associations between EMT gene expression and smoking status (smoker *vs* non-smoker) in a log scale. The top and bottom of the box plot represents the 25th and 75th percentile range. The line across the box shows the median of gene expression and the top and bottom bars show the maximum and minimum values, outliers were showed. The association between EMT gene expression and smoking status was calculated by Fisher’s exact test. **(A)** *BMP7*mRNA (P=0.027); **(B)** *COL3A1*mRNA (P=0.009); **(C)** *EGFR*mRNA (P=0.027); **(D)** *PLEK2*mRNA (P=0.001) and **(E)** *SNAI2*mRNA (P=0.039).

Supplementary Figure 3 Box plot of the associations between EMT gene expression and lymph node metastasis (absence *vs* presence) in a log scale. The top and bottom of the box plot represents the 25th and 75th percentile range. The line across the box shows the median of gene expression and the top and bottom bars show the maximum and minimum values, outliers were showed. The association between EMT gene expression and lymph node metastasis was calculated by Fisher’s exact test. **(A)** *BMP7* mRNA (P=0.005); **(B)** *CALD1*mRNA (P=0.020); **(C)** *CDH1*mRNA (P=0.001); **(D)** *COL3A1*mRNA (P=0.020); **(E)** *EGFR*mRNA (P=0.005); **(F)** *ERBB3*mRNA (P=0.000); **(G)** *PLEK2*mRNA (P=0.024) and **(H)** *TCF4*mRNA (P=0.001).

Supplementary Figure 4 Box plot of the associations between EMT gene expression and distant metastasis (absence *vs* presence) in a log scale. The top and bottom of the box plot represents the 25th and 75th percentile range. The line across the box shows the median of gene expression and the top and bottom bars show the maximum and minimum values, outliers were showed. The association between EMT gene expression and distant metastasis was calculated by Fisher’s exact test. **(A)** *COL3A1*mRNA (P=0.013) and **(B)** *COL5A2*mRNA (P=0.038).
