# Supplementary figures and images for: Pulmonary Neuroendocrine Neoplasms Overexpressing Epithelial-Mesenchymal Transition Mechanical Barriers Genes Lack Immune-Suppressive Response and Present an Increased Risk of Metastasis
Source: Front Oncol. 2021 Aug 30;11:645623. doi: 10.3389/fonc.2021.645623 (PMC8435885; doi:10.3389/fonc.2021.645623)

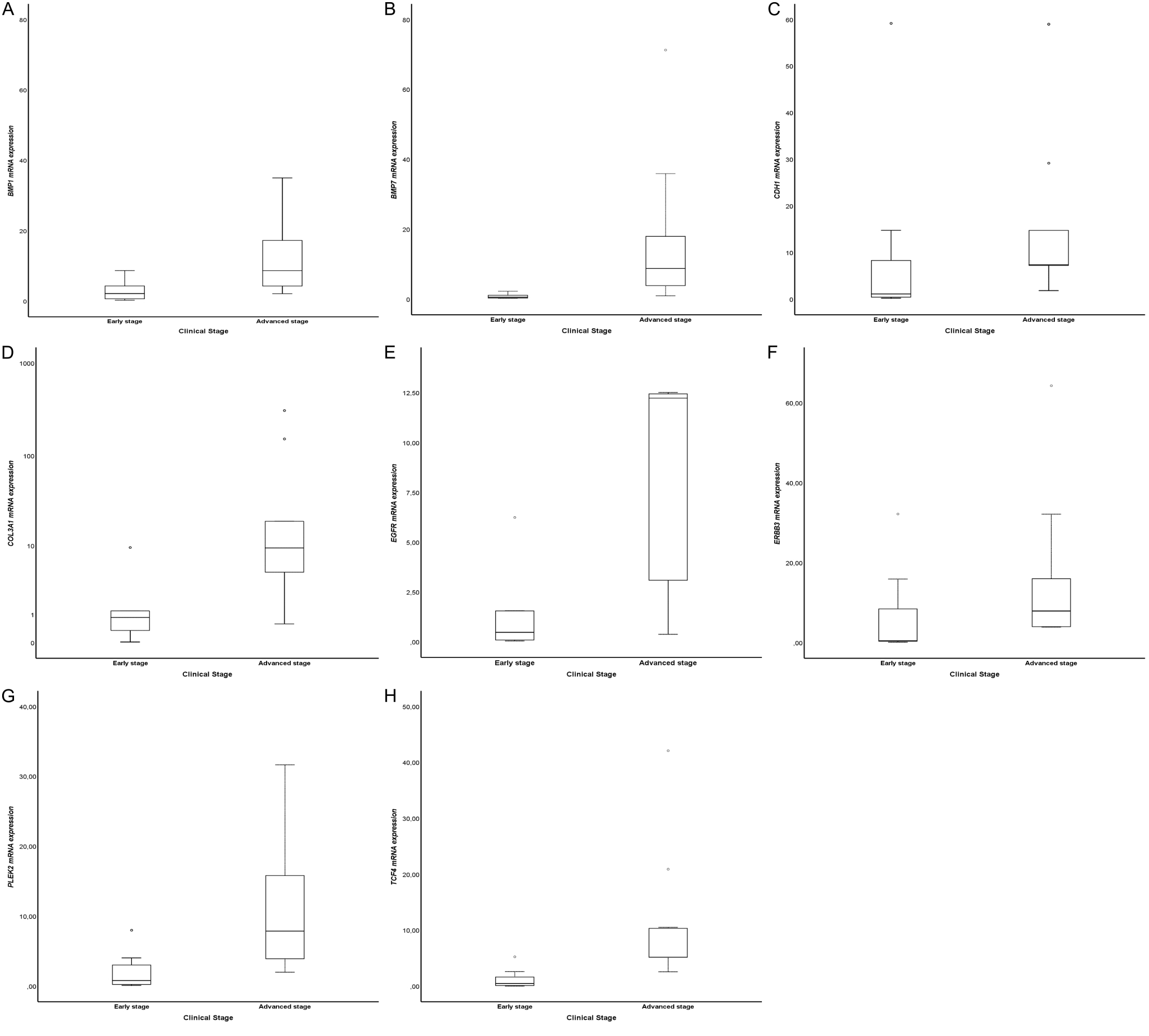

Supplement: Supplementary Figure 1 — Box plot of the associations between EMT gene expression and Clinical stage – Early (I/II) vs Advanced (III/IV) in a log scale. The top and bottom of the box plot represents the 25th and 75th percentile range. The line across the box shows the median of gene expression and the top and bottom bars show the maximum and minimum values, outliers were showed. The association between EMT gene expression and Clinical stage was calculated by Fisher’s exact test. (A) BMP1mRNA (P=0.042); (B) BMP7mRNA (P=0.008); (C) CDH1mRNA (P=0.002); (D) COL3A1mRNA (P=0.002); (E) EGFR mRNA (P=0.008); (F) ERBB3mRNA (P=0.001); (G) PLEK2mRNA (P=0.003) and (H) TCF4mRNA (P=0.003). [file Image_1.jpeg]

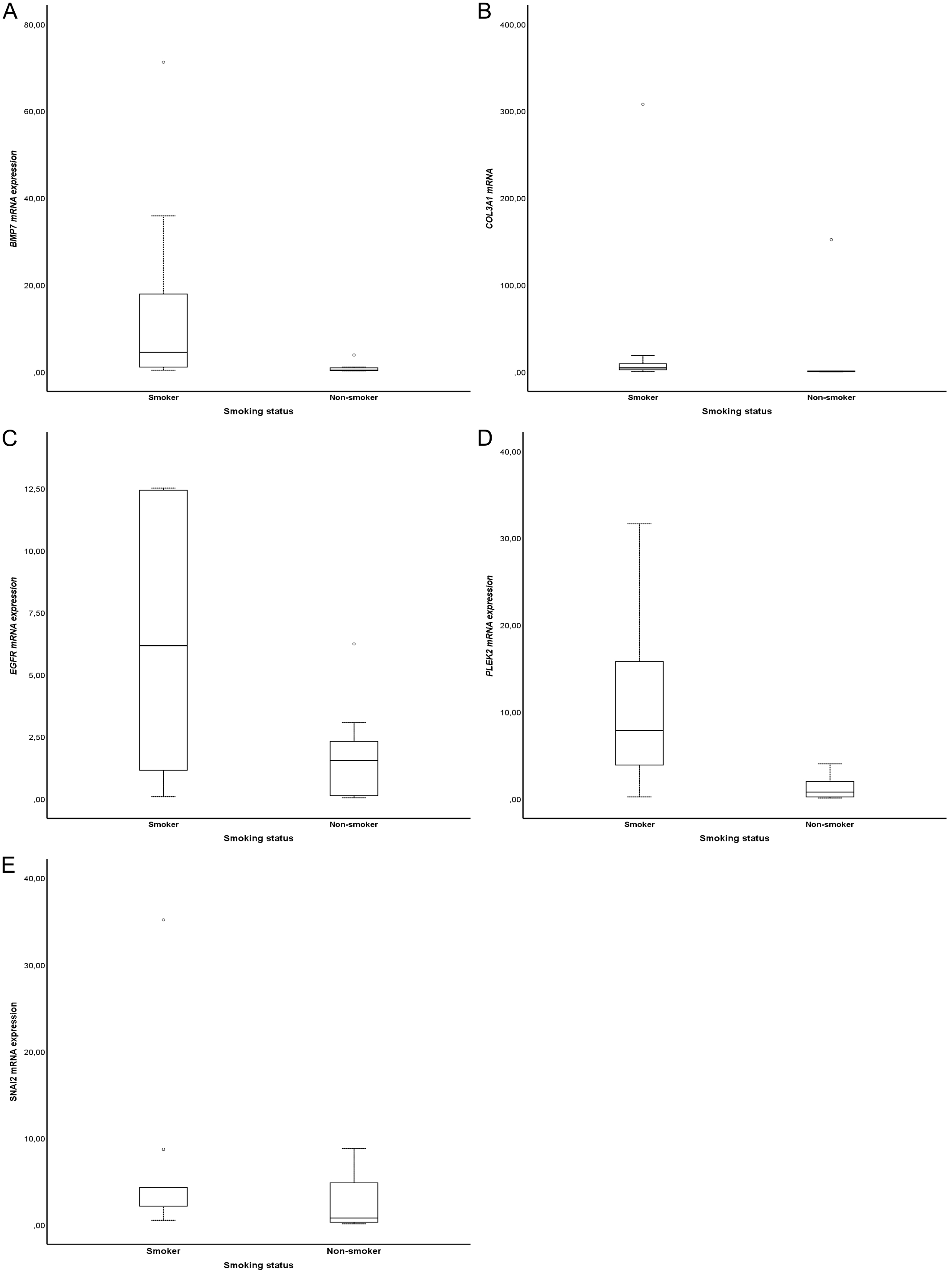

Supplement: Supplementary Figure 2 — Box plot of the associations between EMT gene expression and smoking status (smoker vs non-smoker) in a log scale. The top and bottom of the box plot represents the 25th and 75th percentile range. The line across the box shows the median of gene expression and the top and bottom bars show the maximum and minimum values, outliers were showed. The association between EMT gene expression and smoking status was calculated by Fisher’s exact test. (A) BMP7mRNA (P=0.027); (B) COL3A1mRNA (P=0.009); (C) EGFRmRNA (P=0.027); (D) PLEK2mRNA (P=0.001) and (E) SNAI2mRNA (P=0.039). [file Image_2.jpeg]

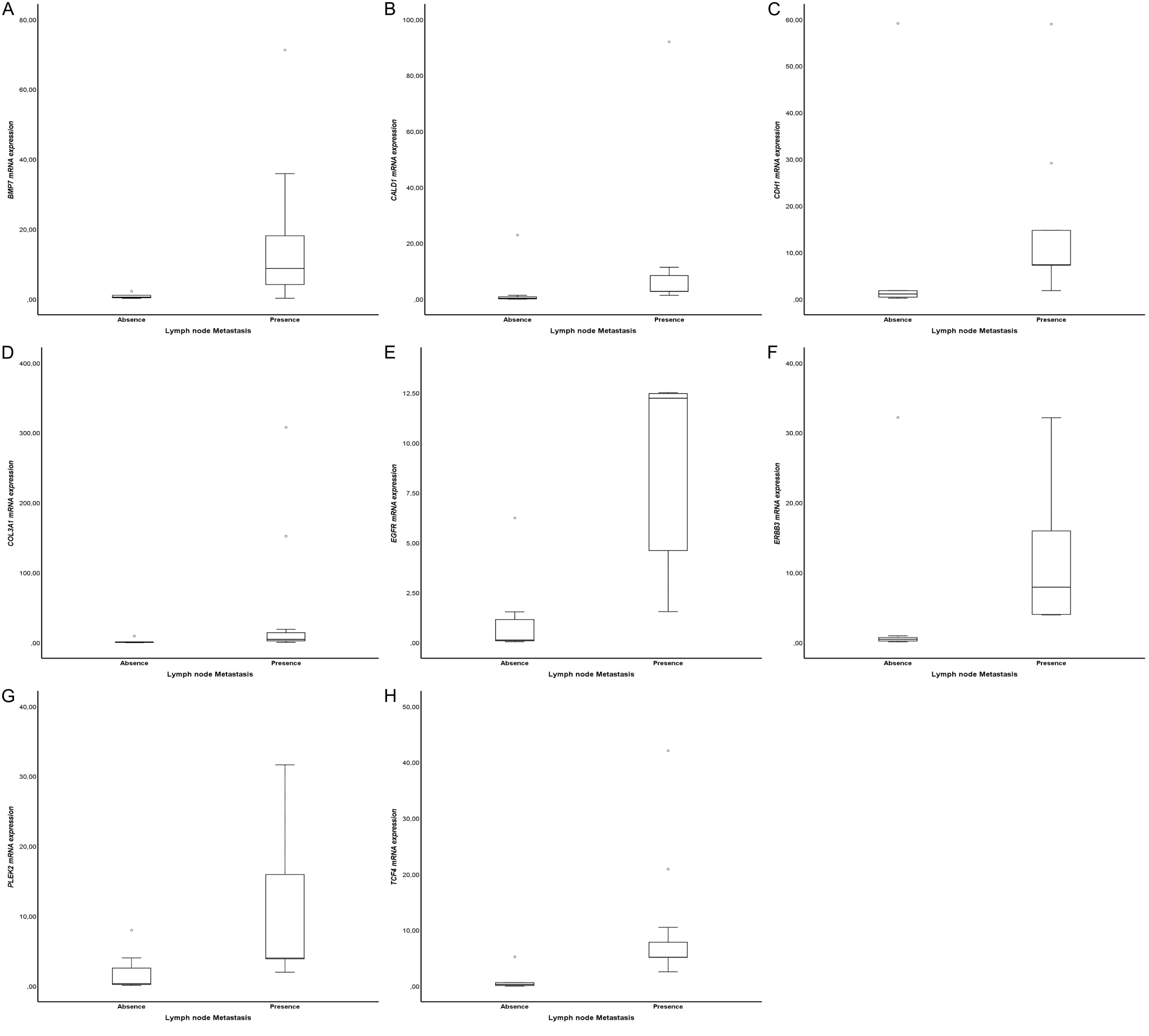

Supplement: Supplementary Figure 3 — Box plot of the associations between EMT gene expression and lymph node metastasis (absence vs presence) in a log scale. The top and bottom of the box plot represents the 25th and 75th percentile range. The line across the box shows the median of gene expression and the top and bottom bars show the maximum and minimum values, outliers were showed. The association between EMT gene expression and lymph node metastasis was calculated by Fisher’s exact test. (A) BMP7 mRNA (P=0.005); (B) CALD1mRNA (P=0.020); (C) CDH1mRNA (P=0.001); (D) COL3A1mRNA (P=0.020); (E) EGFRmRNA (P=0.005); (F) ERBB3mRNA (P=0.000); (G) PLEK2mRNA (P=0.024) and (H) TCF4mRNA (P=0.001). [file Image_3.jpeg]

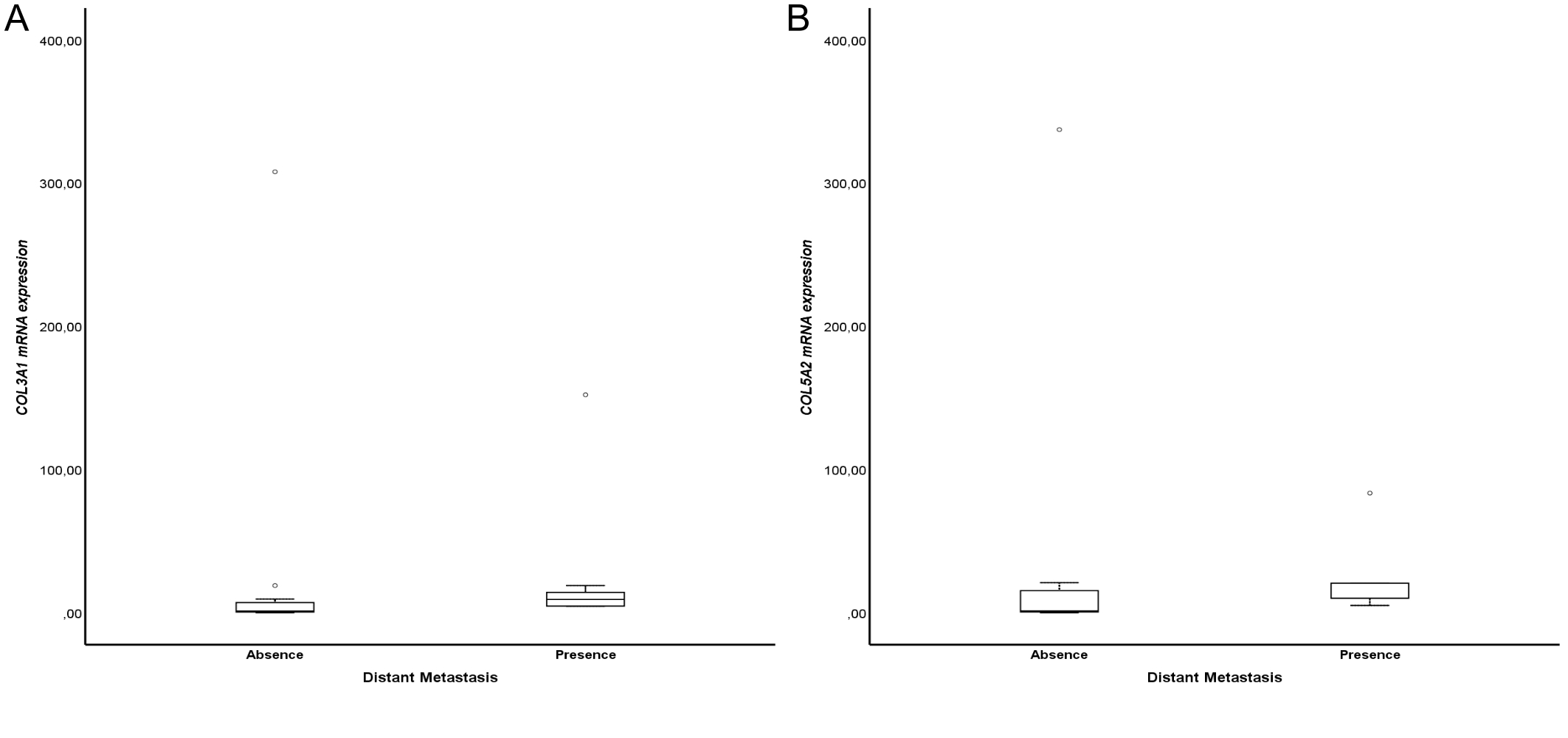

Supplement: Supplementary Figure 4 — Box plot of the associations between EMT gene expression and distant metastasis (absence vs presence) in a log scale. The top and bottom of the box plot represents the 25th and 75th percentile range. The line across the box shows the median of gene expression and the top and bottom bars show the maximum and minimum values, outliers were showed. The association between EMT gene expression and distant metastasis was calculated by Fisher’s exact test. (A) COL3A1mRNA (P=0.013) and (B) COL5A2mRNA (P=0.038). [file Image_4.jpeg]
