# Supplementary material for: Pulmonary Neuroendocrine Neoplasms Overexpressing Epithelial-Mesenchymal Transition Mechanical Barriers Genes Lack Immune-Suppressive Response and Present an Increased Risk of Metastasis
Source: Front Oncol. 2021 Aug 30;11:645623. doi: 10.3389/fonc.2021.645623 (PMC8435885; doi:10.3389/fonc.2021.645623)
Supplement: Supplementary file 5 [file Table_1.docx]

|  | **1** | **2** | **3** | **4** | **5** | **6** | **7** | **8** | **9** | **10** | **11** | **12** |
| --- | --- | --- | --- | --- | --- | --- | --- | --- | --- | --- | --- | --- |
| **A** | AHNAK | AKT1 | BMP1 | BMP2 | BMP7 | CALD1 | CAMK2N1 | CAV2 | CDH1 | CDH2 | COL1A2 | COL3A1 |
| **B** | COL5A2 | CTNNB1 | DSC2 | DSP | EGFR | ERBB3 | ESR1 | F11R | FGFBP1 | FN1 | FOXC2 | FZD7 |
| **C** | GNG11 | GSC | GSK3B | IGFBP4 | IL1RN | ILK | ITGA5 | ITGAV | ITGB1 | JAG1 | KRT14 | KRT19 |
| **D** | KRT7 | MAP1B | MMP2 | MMP3 | MMP9 | MSN | MST1R | NODAL | NOTCH1 | NUDT13 | OCLN | PDGFRB |
| **E** | PLEK2 | DESI1 | PTK2 | PTP4A1 | RAC1 | RGS2 | SERPINE1 | GEMIN2 | SMAD2 | SNAI1 | SNAI2 | SNAI3 |
| **F** | SOX10 | SPARC | SPP1 | STAT3 | STEAP1 | TCF3 | TCF4 | TFPI2 | TGFB1 | TGFB2 | TGFB3 | TIMP1 |
| **G** | TMEFF1 | TMEM132A | TSPAN13 | TWIST1 | VCAN | VIM | VPS13A | WNT11 | WNT5A | WNT5B | ZEB1 | ZEB2 |
| **H** | ACTB | B2M | GAPDH | HPRT1 | RPLP0 | HGDC | RTC | RTC | RTC | PPC | PPC | PPC |

Suppl. Table S1. Genes enrolled in the RT^2^ Profiler PCR array human epithelial mesenchymal transition.

RTC - Reverse Transcription Control; PPC - Positive PCR Controls.
